# High-dynamic-range micro-CT for nondestructive testing of titanium 3D-printed medical components

**DOI:** 10.1117/1.JMI.9.4.044004

**Published:** 2022-08-01

**Authors:** Santiago Fabian Cobos, Christopher James Norley, Steven Ingo Pollmann, David Wayne Holdsworth

**Affiliations:** a University of Western Ontario, Schulich School of Medicine and Dentistry, Department of Medical Biophysics, London, Ontario, Canada; b University of Western Ontario, Robarts Research Institute, Imaging Research Laboratories, London, Ontario, Canada

**Keywords:** high-dynamic-range radiography, x-ray imaging, nondestructive testing, additive manufacturing, micro-CT imaging, laser powder bed fusion

## Abstract

**Purpose:**

Industrial microcomputed tomography (micro-CT) scanners are suitable for nondestructive testing (NDT) of metal, 3D-printed medical components. Typically, these scanners are equipped with high-energy sources that require heavy shielding and costly infrastructure to operate safely, making routine NDT of medical components prohibitively expensive. Alternatively, fixed-current, low-cost x-ray units could be implemented to perform CT-based NDT of 3D-printed medical parts in a subset of cases, if there is sufficient x-ray transmission for the CT reconstruction. A lack of signal—caused by areas of high attenuation in two-dimensional-projection images of metal objects—leads to artifacts that can make an image-based NDT unreliable. We present the implementation of a dual-exposure technique devised to extend the dynamic range (DR) of a commercially available CT scanner equipped with a low-cost low-energy (80 kV) x-ray unit, increasing the signal-to-noise ratio of highly attenuated areas for NDT of 3D-printed medical components.

**Approach:**

Our high-dynamic-range CT (HDR-CT) technique adequately combines projection images acquired at two exposure levels by modifying the integration times of each protocol. We evaluate the performance and limitations of this HDR-CT technique by imaging a series of titanium-alloy test-samples. One of the test-samples was a resolution and conspicuity phantom designed to assess the improvements in void visualization of the proposed methodology. The other test-samples were four porous cylinders, 17×40  mm, with 60%, 70%, 80%, and 90% nominal internal porosities.

**Results:**

Our HDR-CT technique adequately combines projection images acquired at two exposure levels by modifying the integration times of each protocol. Our results demonstrate that the 12-bit native DR of the CT scanner was increased to effective values of between 14 and 16 bits.

**Conclusions:**

The HDR-CT reconstructions showed improved contrast-to-noise and void conspicuity, when compared with conventional CT scans. This extension of DR has the potential to improve defect visualization during NDT of medium-size, titanium-alloy, 3D-printed medical components.

## 1 Introduction

Industrial micro-computed tomography (micro-CT) scanners are an effective tool for nondestructive testing (NDT) of parts fabricated for the automotive, aerospace, pharmaceutical, and medical industries.^1^^,^^2^ However, in some cases, routine NDT using these scanners can become prohibitively expensive.^3^ This is mainly due to the relatively high cost of these scanners that are equipped with high-energy x-ray sources (160 to 450 kV), heavy x-ray shielding, and sophisticated additional hardware. The Nikon XT H 225 industrial scanner is an example of such a system, weighing in at more than 2400 kg and costing more than $750,000. In this system, NDT of a medium-sized, titanium, and 3D-printed part will typically cost over 50% of the manufacturing cost of the part.

Advancements in metal additive-manufacturing, more specifically, laser powder bed fusion (LPBF), have enabled the manufacturing of complex, biocompatible, and mechanically advantageous medical components.^4^ These biocompatible components have common physical characteristics that make them more suitable for image-based NDT using x-ray sources at lower—and thereby more cost-effective—energies (80 kV). These characteristics include small-to-medium size, relatively low-attenuation biocompatible metals, porous constructs, and organic geometries or natural shapes. In most cases, these characteristics reduce the required x-ray penetration to resolve internal features in the part when using CT techniques. However, in other cases, the design of the part might include large portions of solid metal, making low-energy NDT unreliable.

The objects with large portions of solid metal are difficult to interrogate using a fixed x-ray exposure, due to the wide range of intensities over the full field-of-view. In these cases, the imaging parameters (i.e., exposure time, kV, and tube current) are optimized so the exposure does not exceed the dynamic range (DR) of the x-ray detector. As a result, the areas of high-attenuation may become obscured in two-dimensional projection images and will present an insufficient signal-to-noise ratio (SNR) —leading to photon starvation and “under-ranging” artifacts in the CT reconstruction. If the exposure time and/or tube current were increased to achieve sufficient SNR in these high-attenuating areas, the regions of low-attenuation will exceed the DR of the detector (i.e., will be saturated), which will preclude accurate CT reconstruction.

To address this issue, several approaches have been proposed. Most involve hardware modifications to the x-ray detector, which are intended to extend the DR of the device. For example, a detector could include multiple sensors, with different x-ray sensitivity, inside a single pixel.^5^ These specialized detectors could also be manufactured with special scintillators and pixel designs.^6^ Alternatively, software-based methods have also been proposed, in which case, the detector technology does not need to be altered. These techniques to extend the DR of optical detectors are based on the acquisition of multiple images with different x-ray energies or exposure levels.^7^^–^^13^ In both cases, at least two images—one with high-energy/exposure and one with low-energy/exposure—are combined to generate a composite, high-dynamic-range (HDR) projection.^14^ If appropriately combined, these projection images can be used to reconstruct volumetric data with improved image quality and diminished CT image artifacts.

Previously described, dual-exposure HDR-CT methods have been designed for imaging of samples made of various materials with relatively high differences in x-ray attenuation, at a lower energy (50 kV), and multiple tube currents (mA).^9^ Therefore, it is still unclear if the same methodology can be used to improve the image quality of CT-based NDT of solid medical-grade alloys using an 80-kV x-ray energy and a fixed-current (mA) protocol. It is also unclear what the limitations of fixed mA, dual-exposure HDR-CT will be, as a method to diminish photon-starvation artifacts when imaging biomedical alloys.

In this study, we describe the first implementation of lower-energy (80 kV), dual-exposure HDR-CT for NDT of medical components fabricated in medical-grade, titanium-alloy using an LPBF. We describe the hardware and software required to acquire and process HDR projection data and to obtain HDR-CT reconstructions using a commercially available, micro-CT scanner. We demonstrate the advantages of this technique by inspecting a series of 3D-printed, 17-mm diameter, cylindrical, and porous scaffolds designed to have a range of internal porosity fraction (e.g., 60%, 70%, 80%, and 90%). Finally, we evaluate the performance limits of this technique by imaging a customized 3D-printed titanium-alloy resolution phantom, which was designed to evaluate the penetration limits of HDR-CT for NDT of parts fabricated in this commonly used medical-grade alloy.

## 2 Methods

### 2.1 Scanned Test Samples

**Fig. 1:**
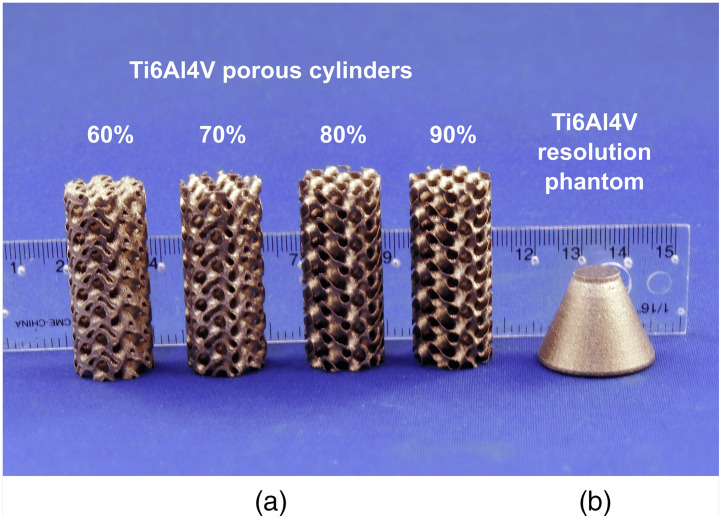
Test samples scanned in this project. (a) Titanium-alloy, gyroid-based, and cylindrical samples with porosity fractions of 60%, 70%, 80%, and 90%. (b) Titanium-alloy, resolution, and conspicuity phantom. The internal features of this resolution phantom are shown in Fig. 2.

Figure 1 shows the five test samples scanned for this study. They were manufactured in titanium-alloy (Ti6Al4V ELI-0406, Renishaw plc, United Kingdom, particle size 15 to 45μm) using an LPBF 3D-printer for metal alloys (AM400, Renishaw plc, Wotton-under-Edge, United Kingdom) at the Additive Design in Surgical Solutions facility (ADEISS) in London, Canada.

Four objects were designed as cylinders, 17 mm in diameter and 40 mm in length, with nominal internal porosities of 60%, 70%, 80%, and 90%. The internal porosity of these cylindrical test objects was achieved by modifying the thickness of a ${6\text{-}\mathrm{mm}}^{3}$, sheet-based gyroid unit. The computer-assisted design (CAD) for these test objects was performed in Blender (Version 2.79, blender.org, Amsterdam, The Netherlands).

**Fig. 2:**
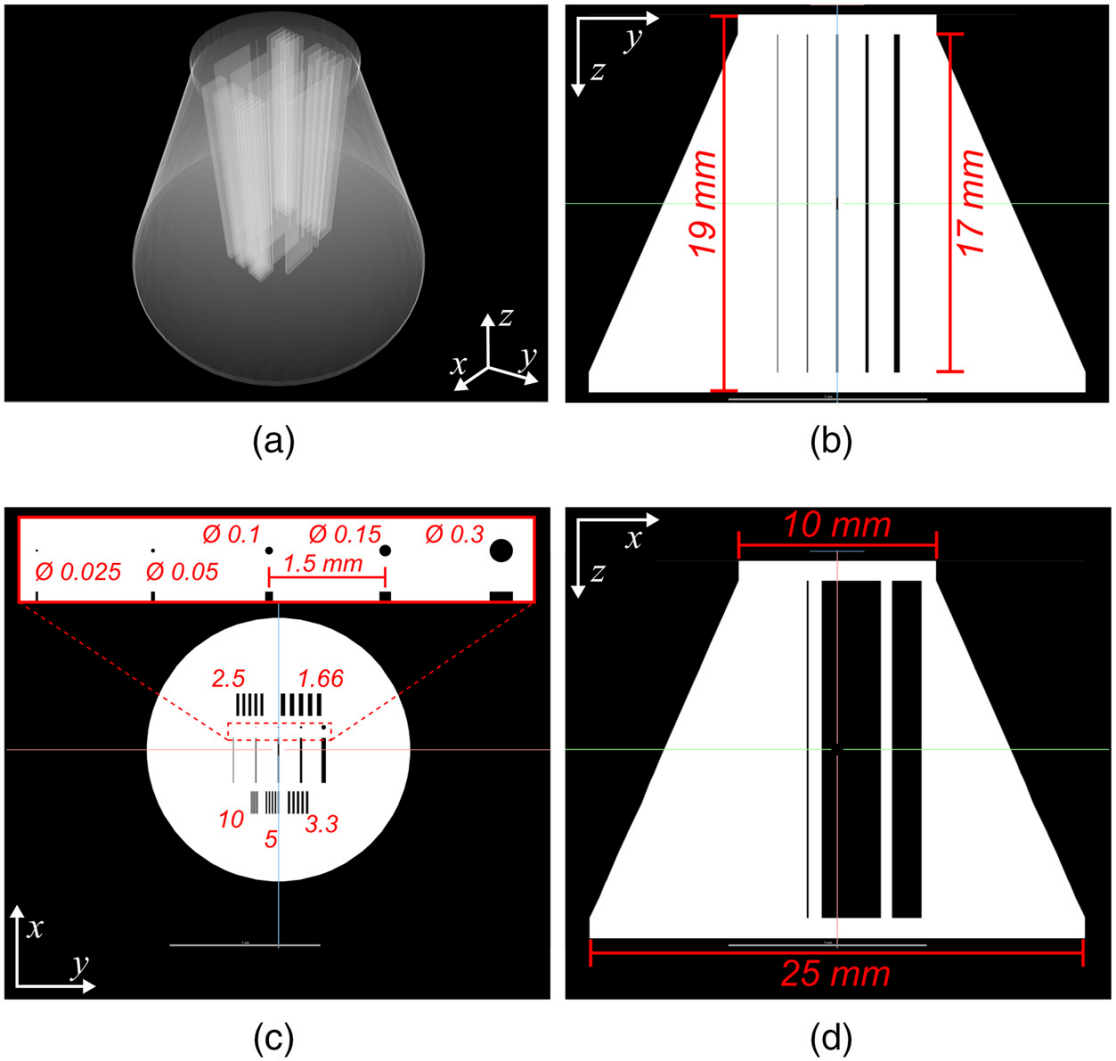
Detailed schematics of the titanium-alloy, resolution, and conspicuity phantom. (a) Perspective view of the 3D-rendering of the CAD with a transparency filter. (b) Central, trans-coronal, synthetic slice of the phantom’s CAD. (c) Central, trans-axial, synthetic slice of the phantom’s CAD, showing the details of the internal voids. The bar patterns are described in lp ${\mathrm{mm}}^{-1}$. All other measurements are shown in mm. (d) Central, trans-sagittal synthetic slice of the phantom’s CAD.

The other test sample was designed as a truncated cone, 17 mm high, with a 25-mm-diameter base and a 10-mm diameter top. Internally, it incorporated several series of deliberately prescribed voids of various widths, corresponding to a range of line-pairs (1.6 to $10\;{\mathrm{mm}}^{-1}$), which extended from the top of the truncated cone to its base, along its longitudinal (i.e., central) axis. It also incorporated a series of voids of various widths, centrally located, to determine void conspicuity. This resolution phantom was designed to evaluate the ability of the CT reconstructions to resolve known internal features of various sizes within the object as the cumulative x-ray path-length through the metal increases. Figure 2 shows the detailed schematics of this titanium-alloy resolution and conspicuity phantom.

### 2.2 Dual-Exposure Data Acquisition

Four image acquisition protocols with increasing integration times were designed to image the titanium-alloy, 3D-printed samples for this study. The micro-CT scanner (eXplore Locus RS x-ray, GE Healthcare; London, Canada) operated at a peak tube potential of 80 kV and a fixed tube current of 400  μA. The first protocol had an integration time of 261.3 ms, which was optimized for the 12-bit x-ray detector, so in an unattenuated region of the field-of-view the intensity values for each pixel did not exceed the maximum value of the detector [i.e., intensity $<2^{12}=4096$ analog-to-digital units (ADU)] of the eXplore Locus RS scanner. Unfortunately, it also meant that, during data acquisition, the pixels obscured by highly attenuating regions on the image were underexposed. The integration time of the other three protocols was designed to have nominally 4, 8, and 16 ($2^{2},2^{3}$, and $2^{4}$, respectively) times the exposure of the first protocol. For simplicity, in this paper, we will refer to these protocols using the exposure value (EV) bracketing convention that is common to HDR optical photography. Here, EV0 represents the nonsaturated, short acquisition that is optimized for the 12-bit detector, and EVn represents acquisitions with exposures that are nominally “$2^{n}$” times the EV0 exposure. Table 1 summarizes the acquisition parameters for each EV protocol at 80 kV and 400  μA.

**Table 1:** Dual-exposure HDR protocols for the eXplore Locus RS x-ray scanner.

	EV0	EV2	EV3	EV4
Prescribed integration-time (ms)	200	981	2026	4116
True integration-time (ms)	261.3	1044	2090.6	4182.3
EVn-to-EV0 exposure ratio	1	4	8	16
Exposure value/bracketing (f-stop equivalent)	0	2	3	4

The integration times prescribed into the scanner’s control interface differed from the true integration times. X-ray exposure times were measured with a custom sensor timer circuit, comprised of a phototransistor (APDS-9930) in contact with a small phosphor scintillator (Lanex regular); timing was achieved with an interrupt-driven microprocessor (Arduino Uno). It was empirically observed that true integration times were, on average, 65 ms longer than the nominal prescribed times in the protocol. Therefore, the prescribed integration times for EV0, EV2, EV3, and EV4 were revised to be 200, 981, 2026, and 4116 ms, respectively.

For each protocol, the objects were placed in the field-of-view of the scanner using a custom-made holder to circumvent the rehoming of the specimen loading bed of the eXplore Locus RS scanner. This precaution was implemented because it was determined that the linear bed mechanism was not able to return to the initial scanning position with sufficient accuracy, which resulted in registration errors when combining projection images for HDR radiography.

Each protocol, starting at EV0, collected a total of 200 projection images at 1-deg angular increments. For each subsequent EV protocol, the projection images were inspected to ensure that the EVn images were not completely saturated and that they included at least 50% of the sample’s valid data. Dark-field images, required for offset correction, for each protocol were acquired in a way that matched that of each EV condition. Finally, only one bright-field image was acquired using the EV0 protocol, and this bright-field image was scaled appropriately for use with each HDR-CT reconstruction.

### 2.3 Dual-Exposure HDR Projection Data Postprocessing

HDR projection images were generated by combining pairs of EV0 and EVn data. Dark noise in each image of the pair was corrected using dark fields (i.e., images acquired with no x-rays on) acquired with their respective EV0 and EVn integration times. This was necessary to address the time-dependent aspect of dark-noise intensity. Next, the EV0 image was multiplied by a scaling factor (EVnf) to increase signal values it to match EVn. The scaling factor was calculated on a per-projection basis using the following equation:

$$\begin{array}{c} \mathrm{EVnf}=\frac{I_{{\mathrm{EV}}_{n}}}{I_{\mathrm{EV}0}}\; \end{array},$$ (1)

where $I_{\mathrm{EV}0}$ and $I_{\text{EVn}}$ are the mean intensity of the valid data that were selected using a mask that included only pixels with intensities <95% of the saturation value in EVn, prior to dark-noise correction. For each pair, the dark-corrected, bright-field image acquired using the EV0 protocol was multiplied by the average of the set of EVnf values to synthesize a bright-field for the EVn acquisition. This “synthetic” bright-field image was later used to flat-field (i.e., gain) correct the HDR projection images and to calculate the per-pixel $I_{0}$ values required for the CT reconstruction.

The final step was to combine the dark-noise-corrected and intensity-matched EV0 (IM EV0) projection images with the corresponding set of dark-noise-corrected EVn projections, using only valid data optimized from each. From visual inspection of thresholded EVn projections, it was determined that the EVn data were valid below 95% of the saturation value in those images. Similarly, for the intensity-matched EV0 projections, it was determined that regions in the EV0 images below the 75% saturation value were underexposed due to photon starvation at the detector and should thus be discarded. Thus, the pixels in EV0 image above the 75% saturation value were considered valid. For pixels values between these two thresholds ($t_{95}$ and $t_{75}$), where both EV0 and EVn contained valid data, their intensities ($I_{x,y}$) were assigned using a weighted average using the following equation:

$$\begin{array}{c} I_{x,y}=(\sin(\frac{\mathrm{EV}0_{x,y}-t_{95}}{t_{95}-t_{75}}){)}^{2}\mathrm{EV}0_{x,y}+\;(\cos(\frac{\mathrm{EV}0_{x,y}-t_{95}}{t_{95}-t_{75}}){)}^{2}{\mathrm{EVn}}_{x,y}. \end{array}\;$$ (2)

**Fig. 3:**
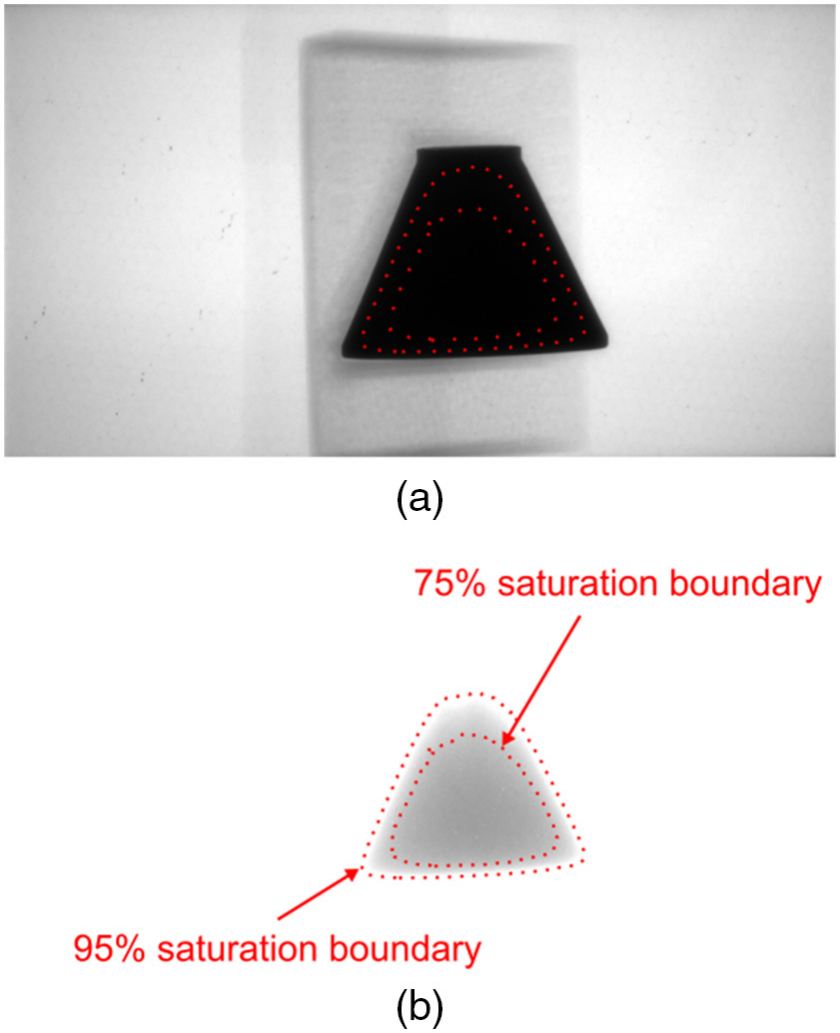
Projection images used to generate HDR data showing the location of the 95% saturation and 75% saturation threshold boundaries. (a) Projection image of the titanium-alloy, resolution phantom acquired using the EV0 protocol. (b) Projection image of the titanium-alloy, resolution phantom acquired using the EV4 protocol.

This $\sin^{2}+\cos^{2}$ functional combination was chosen to minimize image stitching artifacts between the two data sets and to reduce the likelihood of discontinuity artifacts in the CT reconstructions, which have been described in the literature as a common limitation of HDR-CT.^9^^,^^15^ Figure 3(a) shows the location of the threshold boundaries in an EV0 projection image of the titanium-alloy resolution phantom, and Fig. 3(b) shows the same boundaries in a EV4 projection image.

The final postprocessing step, before CT reconstruction, involved the implementation of a combined, wavelet-Fourier, ring-artifact removal filter, following the protocol described by Münch et al.^16^ This additional processing step was required to compensate for the reduced efficacy of the gain-and-offset correction achieved using the intensity-matched bright-field only.

### 2.4 CT Reconstruction and Data Analysis

The 200 projection images collected with the EV0 protocol and each of the HDR pairs (EV0 and EVn) was used to perform CT reconstructions using a limited-view (i.e., 200 deg angular range), Parker-weighted, Feldkamp, Davis, and Kress, filtered-backprojection algorithm.^17^ The voxel spacing of the reconstruction matrix was 90×90×90  μm. Prior to CT reconstruction, the projection images for the cylindrical samples were corrected for beam-hardening using the protocol described by Edey et al.^18^ Note that it was not possible to implement this correction in the reconstruction of the resolution phantom, because the phantom diameter exceeded the thickness of the calibration object used for the beam-hardening correction. Furthermore, the correction parameters were different for each HDR image set, which made it difficult to compare the improvements in conspicuity with increasing penetration depth in this phantom that were due to HDR-CT alone.

Improvements in conspicuity with increasing penetration depth were assessed using the titanium-alloy resolution and conspicuity phantom shown in Fig. 2. Conspicuity was qualitatively evaluated using a trans-coronal CT slice plane perpendicular to the prismatic voids located inside the central region of the phantom—similar to the synthetic slice shown in Fig. 2(b). Visualization window and level were optimized by visual inspection to depict the best contrast for the EV0 reconstruction, which was compared with the HDR pairs.

The cylindrical test samples were used to evaluate the HDR-CT technique for highly porous geometries. These reconstructions were visually inspected in trans-sagittal CT slices to visualize any discontinuity artifacts caused by the HDR stitching. These slices were also interrogated using a line-profile across the walls of the gyroid unit to evaluate improvements in image quality and SNR between the conventional-DR and the HDR volumes.

## 3 Results

### 3.1 Generation of HDR Projection Images

HDR projection images were successfully generated for all test samples, with the exception of the 90% porous cylinder, due to the high degree of overexposure of the projection images for protocols with integration times longer than that of EV0. Table 2 provides a summary of the EV0 and EVn pairs used to generate HDR-CT reconstructions for each test sample. The 12-bit DR (4096 ADU) of the detector was effectively extended to 14.18 (18,514 ADU), 15.24 (38,625 ADU), and 16.29 bits (80,118 ADU) for EV2, EV3, and EV4, respectively.

**Table 2:** Summary of HDR-CT protocol-pairs per test sample.

	Conventional dynamic range	EV0–EV2	EV0–EV3	EV0–EV4
Dynamic range (hardware and effective)	4096 ADU (12-bit detector)	18,514 ADU (14.18 bits)	38,625 ADU (15.24 bits)	80,118 ADU (16.29 bits)
90% porous cylinder	Yes	No	No	No
80% porous cylinder	Yes	Yes	No	No
70% porous cylinder	Yes	Yes	Yes	No
60% porous cylinder	Yes	Yes	Yes	No
Resolution phantom	Yes	Yes	Yes	Yes

The intensity of the pixels (${\mathrm{HDR}}_{x,y}$) of an HDR EV0 and EVn projection image pair was assigned following three conditions:

• If the pixel intensity in EVn, prior to dark correction, was >95% of the saturation value, then it was categorized as invalid, and therefore, was assigned the value of the intensity-matched, dark-corrected EV0 image.
• If the pixel intensity value in EVn, prior to dark correction, was <75% of the saturation value, then it was categorized as valid, and therefore, was assigned the value of the dark-corrected EVn image.
• If the pixel intensity value in EVn, prior to dark correction, was in between 75% and 95% of the saturation value, then it was categorized as valid in both EV0 and EVn images, and therefore, was assigned a weighted-average value using the $\sin^{2}+\cos^{2}=1$ identity, following Eq. (2).

**Fig. 4:**
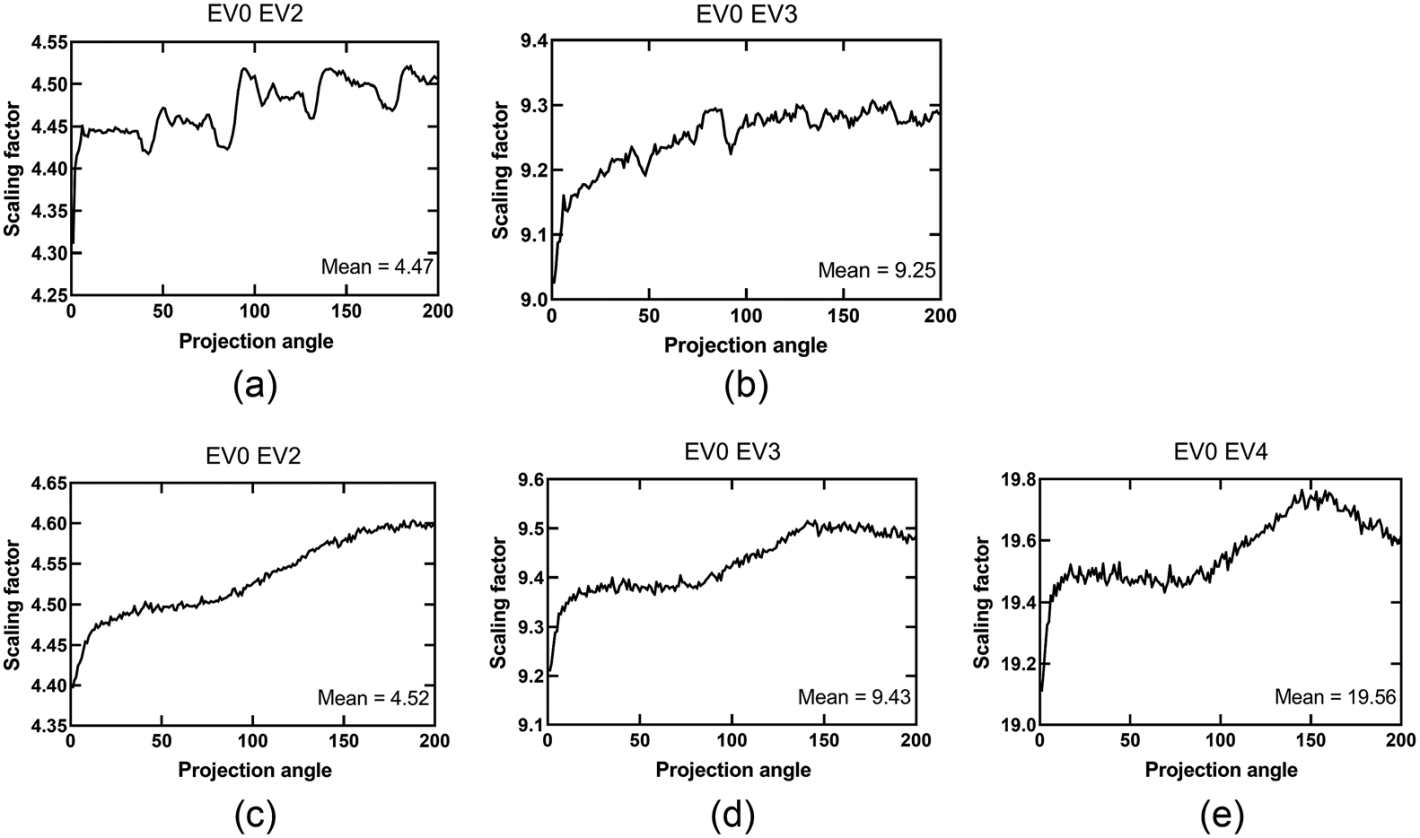
Scaling factors [Eq. (1)] for matching the intensity of EV0 projection images to EVn as a function of projection angle for the 60% porosity cylinder for: (a) the EV0, EV2 HDR pair and (b) the EV0, EV3 HDR pair, and for the resolution and conspicuity phantom for: (c) the EV0, EV2 HDR pair; (d) the EV0, EV3 HDR pair; and (e) the EV0, EV4 HDR pair.

Figure 4 shows angle-dependent variations in the factors used to match EV0 intensity values for the 60% porous cylinder and the resolution and conspicuity phantom to each corresponding EVn on a per-projection basis. These scaling factors showed the same variability between samples, and the variability trend described a specific signature for each object.

**Fig. 5:**
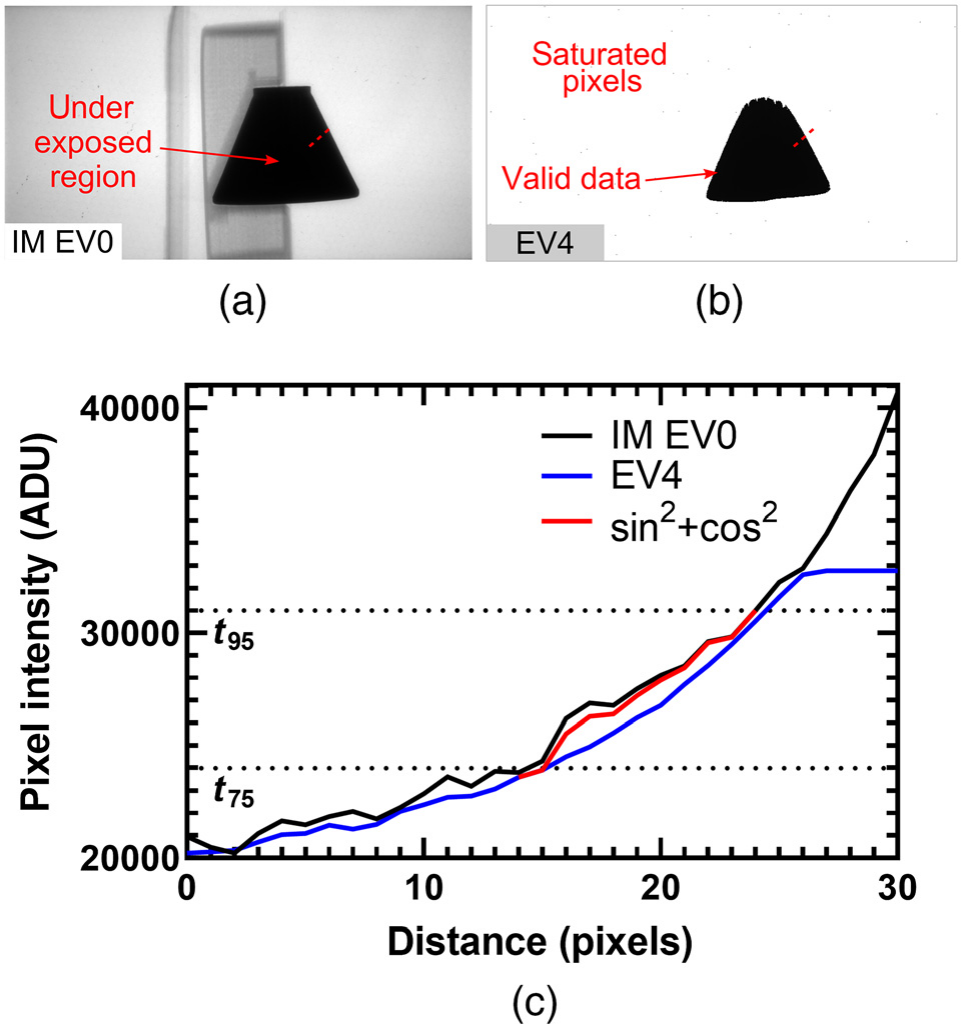
HDR strategy for the combination of EV0 with EVn projection images. (a) Dark-corrected, EV0 projection image of the resolution and conspicuity phantom, scaled to match the intensity values of EV4 (i.e., intensity-matched, IM EV0). Due to a long ray path through the metal in the central region of the phantom, pixels were underexposed in the EV0 image and were discarded from the final HDR projection image. (b) Corresponding, dark-corrected EV4 projection, showing regions with saturated pixels and valid data. (c) Line-profile through pixels marked by the red-dotted line in (a) and (b) showing the $\sin^{2}+\cos^{2}$, weighted-average combination strategy for pixels with intensity values between the 95% ($t_{95}$) and 75% ($t_{75}$) saturation thresholds.

The EV0 and EVn combination strategy, in projection images, demonstrated the ability to remove any visually obvious stitching artifacts. Figure 5 shows the results of this procedure for one of the projection images of the resolution and conspicuity phantom, obtained using the EV0 and EV4 protocols. This figure shows that the EV0 data have been appropriately scaled to closely match the intensity values of EV4 below the 75% saturation threshold ($t_{75}$). Furthermore, the intensity-matched EV0 (IM EV0) data over the 95% saturation threshold ($t_{95}$) properly extrapolate the intensity values of EV4 for the region in EV4 where pixels reach the saturation point. The HDR intensity values in between the $t_{75}$ and $t_{95}$ thresholds were calculated using the previously described $\sin^{2}+\cos^{2}=1$ identity [Eq. (2)]. Figure 5(c) shows the effectiveness of this strategy in the combination of the two datasets, mainly due to the behavior of the identity near the thresholds, which ensured that the combined pixel values were properly weighted between the IM EV0 and the EV4 data. Specifically, the intensity value of a combined HDR pixel was heavily weighted toward EV0 near the $t_{95}$ threshold and heavily weighted toward EV4 near the $t_{75}$ threshold, with a smooth transition.

### 3.2 HDR-CT of Porous Cylindrical Samples

**Fig. 6:**
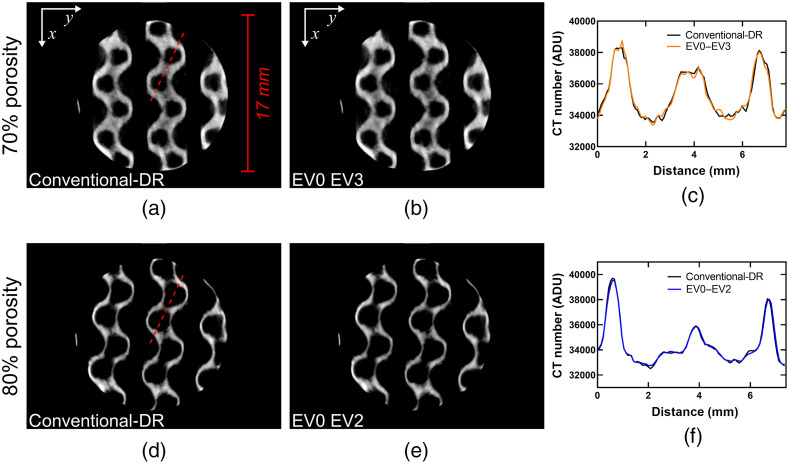
Results of the CT reconstructions for the 60% titanium-alloy, porous cylinder. (a)–(c) A trans-axial slice through the data for the conventional-DR, the EV0 EV2 pair, and the EV0 EV3 pair, respectively. (d) A 3D-rendering of the data in a perspective view. (e) and (f) The voxel values through a line-profile following the red-dotted line in (a) to compare the differences in SNR between datasets.

**Fig. 7:**
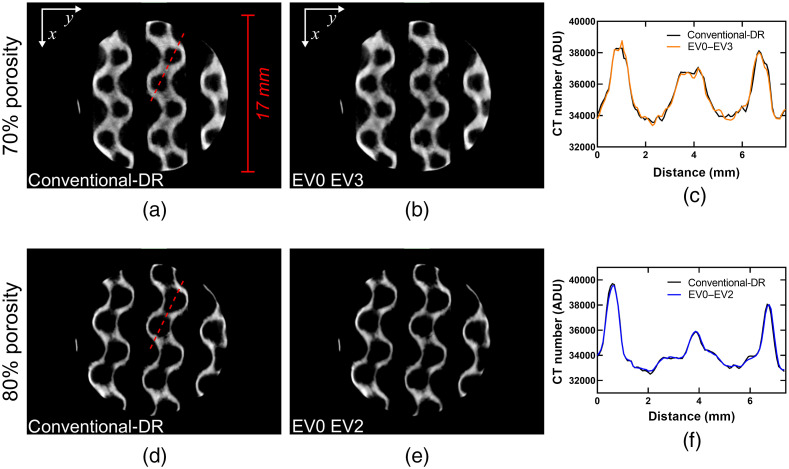
Results of the CT reconstructions for the 70% and 80% titanium-alloy, porous cylinders. (a) and (b) A trans-axial slice through the 70% porous cylinder for the conventional-DR and the EV0 EV3 pair, respectively. (d) and (e) A trans-axial slice through the 80% porous cylinder for the conventional-DR and the EV0 EV2 pair, respectively. (c) and (f) The voxel values through a line-profile following the red-dotted line in (a) and (d) to compare the differences in SNR between the respective datasets.

Figures 6 and 7 show trans-axial slices of the HDR reconstructed volumes using various combinations of EV0 and EVn scans. In all cases, the conventional-DR (i.e., single-exposure) reconstructions exhibit reduced image quality, mainly due to a reduced SNR within the titanium-alloy regions in the conventional-DR reconstruction. For the 60% porous cylinder, the differences in SNR between the EV0 EV2 and the EV0 EV3 HDR reconstructions were not as significant as the differences between the HDR and the conventional-DR volumes.

Figure 7 shows the SNR improvement for the 70% and 80% porous cylinders between the conventional-DR reconstructions and the HDR reconstructions with the longest integration times for each object. The improvements in SNR were more pronounced for the 70% porous cylinder, whereas for the 80% porous cylinder, the improvements in image quality were barely noticeable. These results indicate that for samples with low total x-ray path-lengths through solid metal HDR might not be necessary.

### 3.3 HDR-CT of Resolution Phantom

**Fig. 8:**
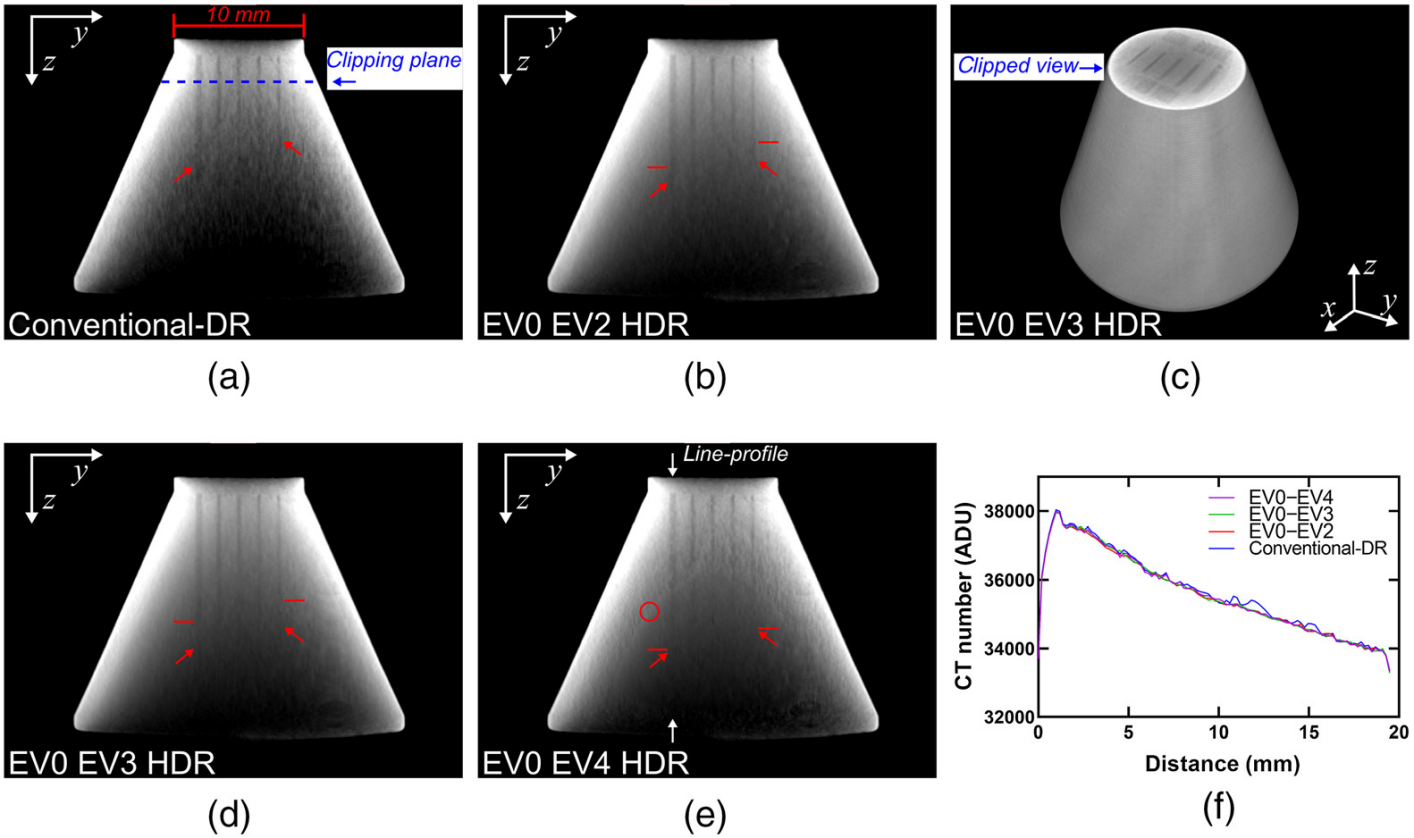
CT reconstructions for the resolution and conspicuity phantom. (a), (b), (d), and (e) A trans-coronal slice through the reconstructed data for the conventional-DR, the EV0 EV2, the EV0 EV3, and EV0 EV4 pairs, respectively. The red arrows in these images describe the location of the void conspicuity limit for the 30- and the 300-μm-wide prismatic voids. The red horizontal lines mark the void conspicuity limit reached by the immediately shorter HDR reconstruction. The red circle in (e) shows the location of a discontinuity artifact for the EV0 EV4 dataset. (c) A 3D rendering of a perspective view of the EV0 EV3 HDR reconstruction with the top portion of the phantom clipped at the level marked in (a). (f) The intensity values of voxels in a profile-line located along the longitudinal axis of the phantom and within the 300  μm wide prismatic void.

The resolution and conspicuity phantom was specifically designed to evaluate the visualization of internal voids for varying total x-ray path-lengths through a block of solid titanium-alloy. This was assessed using a trans-coronal slice placed at the center of the phantom and perpendicular to the prismatic voids that ran through its longitudinal axis. The diameter of the phantom, at the void conspicuity limit for the nominal 30-μm wide prism, was used as a reference to describe the total x-ray path-length limit for each CT reconstruction. The void conspicuity limit was reached at a diameter of 14.45 mm for the conventional-DR reconstruction. The EV0 EV2 HDR reconstruction improved this conspicuity limit up to 16.08 mm and the EV0 EV3 HDR up to 19.84 mm. For the EV0 EV4 reconstruction, further improvements to the void conspicuity limit would be challenging to assess. Unfortunately, the EV0 EV4 reconstruction suffered from artifacts caused by the increased dark-noise collected at this 16× longer exposure. Figure 8(f) shows the improved CT number accuracy in a profile-line through the voxels located along the longitudinal axis of the phantom, and within one of the prismatic voids, which contributes to the observed improvements in void visualization for the HDR-CT reconstructions.

## 4 Discussion

The use of a low-energy (80 kV) x-ray source for imaging-based NDT of medium-sized, titanium-alloy biomedical components is a promising alternative for routine, cost-effective quality control in the additive manufacturing industry. Unfortunately, this solution suffers from limitations due to the low DR of commercially available micro-CT scanners. Here, we demonstrate that NDT can be enhanced by implementing a software-based, dual-exposure HDR radiography technique that requires no hardware modifications to the scanner. This integration-time-dependent HDR strategy takes advantage of the linear response of the x-ray detector, which allows the extension of its DR by modifying the data acquisition process, and strategically combining two images with varying integration times—i.e., a short integration-time EV0, and a long integration-time EVn. We demonstrated that the DR of a micro-CT scanner with a 12-bit detector can be extended up to an effective 16 bits, resulting in CT reconstructions of test samples with improved contrast-to-noise characteristics.

Our HDR method was designed under the constraint that cost-effective, routine NDT is more likely to be achieved using x-ray sources with a relatively low-cost, requiring minimal infrastructure. These x-ray sources operate at lower energies (<80  kV) and at fixed (i.e., maximum) tube currents. For this reason, our implementation of an integration-time-dependent, dual-exposure HDR-CT technique differs from the strategies described by Sisniega et al.^9^ and Chen et al.,^7^ who instead modified the imaging conditions by varying the tube current and tube energy, respectively. Although our results showed that the reconstructions that included data acquired with 4× (EV0 EV2) and 8× (EV0 EV3) the integration time of a conventional-DR CT (EV0) presented improved image quality, for longer integration times, 16× (EV0 EV4), the proposed technique was susceptible to artifacts related to increased dark-noise, image lag, and ghosting. Image lag and ghosting were especially detrimental for cases where the pixel switched from an oversaturated state to linear response from one projection image to the other, which could last up to 4.5 s. Artifacts generated due to image lag and ghosting are likely to be system specific, and some detector technologies might be less susceptible to both phenomena.

For the proposed HDR-CT to be cost-effective, it should only be used in cases where the increase in scan time is advantageous. Data acquisition for the (EV0 EV2) HDR pair takes five times longer than conventional CT images, and the (EV0 EV3) and (EV0 EV4) pairs take 9 and 17 times longer, respectively. One potential solution to prevent prohibitively long scans would be to selectively use HDR imaging only in projection views that require an HDR. This assessment could be done automatically using the EV0 projection data or *a priori* knowledge about the geometry of the object being scanned.

Discontinuity artifacts in HDR-CT reconstructions have been described as one of the main limitations of dual-exposure HDR techniques.^7^^–^^9^^,^^15^ During the development of our methodology, several strategies were tested to combine the EV0 and EVn pairs, but the described $\sin^{2}+\cos^{2}$, weighted-average strategy was the only one to render acceptable results. We believe this is, in part, due to the significant differences in the SNR between the EV0 and the EVn datasets. Any direct stitching between EVn data and intensity-matched EV0 data produced noticeable discontinuity artifacts in the CT reconstructions. Avoiding these discontinuity artifacts was especially challenging for the EV0 EV4 HDR pair, as the interface between valid and overexposed data was always located within the body of the scanned part. For this reason, we do not recommend the acquisition of EVn images where a significant portion of the object in the projection images is overexposed.

Another potential source of these discontinuity artifacts is the incorrect matching of EV0 and EVn intensity values. In our experiments, the best strategy was to calculate the mean intensity of pixels containing valid data in both data sets using a view-angle-dependent approach. Figure 4 shows how the scaling factors varied as a function of projection angle for the resolution and conspicuity phantom data. It was observed that these fluctuations in scaling factors followed a similar trend for the various EVn datasets, but were highly object-dependent. We hypothesize that these fluctuations are caused by the complex relationship between the object geometry and image lag and ghosting artifacts. Fortunately, both of our strategies to prevent discontinuity artifacts yielded good results, with the exception of the EV0 EV4 HDR pair, where artifacts were still conspicuous in some regions of the reconstructed volume.

The improvements in image quality in the HDR-CT reconstructions were derived from a higher SNR in the areas of the image that were otherwise obscured by large volumes of titanium-alloy in our test samples. For instance, in the case of the resolution and conspicuity phantom, HDR-CT increased void conspicuity from an x-ray path-length of 14.45 mm (in conventional-DR) up to 19.8 mm through solid titanium-alloy. This 37% improvement in penetration depth for 80 kV, fixed-current NDT could be a critical improvement for the evaluation of medium-sized medical components that otherwise will suffer from photon-starvation artifacts in regions with a total x-ray path-length over 19.8 mm. Fortunately, most medium-sized, titanium-alloy, 3D-printed medical components are designed with internal geometries with a high-porosity fraction. In such cases, which are similar to the porous cylinders imaged in this study, the degree to which the integration times are extended could be dictated by the density of the object and the path-length through which the x-rays must travel.

## 5 Conclusion

We have successfully implemented a low-energy (80 kV), fixed tube-current HDR-CT strategy for cost-effective, routine NDT of titanium-alloy 3D-printed parts. We were able to generate HDR-CT reconstructions of titanium-alloy 3D-printed samples by successfully combining projection images acquired at two exposure levels, achieved by modifying the integration times of each protocol. These projection images increased the DR of the x-ray detector from its native 12 bits to effective values of 14.18, 15.24, and 16.29 bits. The CT reconstructions generated using these HDR projection images showed improved contrast-to-noise and an improved void visualization, when compared to conventional dynamic-range CT.

Projection images acquired with a short integration time were successfully combined with their long integration-time pairs, using two strategies: (1) by carefully scaling the intensity values of the short-timed exposure in a per-projection-angle basis and (2) by smoothing the transition between data sets across a range of intensity values using an appropriate weighted average.

The proposed exposure-time-dependent, dual-exposure HDR-CT method improved the contrast-to-noise and performance of NDT for medium-sized, titanium-alloy 3D-printed parts. Furthermore, this technique provided a 37% improvement in conspicuity of internal defects in a resolution phantom, in comparison to conventional-DR CT. Future work includes the evaluation of the technique using more clinically relevant geometries, and the investigation of HDR correction for a subset of the projection images, as well as a more rigorous *a priori* determination of the degree to which integration times must be extended for NDT of titanium-alloy, medical components fabricated using additive manufacturing.

## References

[1] De Chiffre L. et al., “Industrial applications of computed tomography,” CIRP Ann. 63(2), 655–677 (2014) CIRAAT 0007-8506 10.1016/j.cirp.2014.05.011

[2] Ewert U. Fuchs T., “Progress in digital industrial radiology. Pt. 2, Computed tomography (CT),” Badania Nieniszczące i Diagnostyka, 7–14 (2017). 10.26357/BNID.2017.018

[3] du Plessis A. le Roux S. G. Guelpa A., “Comparison of medical and industrial x-ray computed tomography for non-destructive testing,” Case Stud. Nondestruct. Test. Eval. 6, 17–25 (2016). 10.1016/j.csndt.2016.07.001

[4] Lowther M. et al., “Clinical, industrial, and research perspectives on powder bed fusion additively manufactured metal implants,” Addit. Manuf. 28, 565–584 (2019). 10.1016/j.addma.2019.05.033

[5] Fox T. et al., “Dynamic range extension of x-ray imaging system used in non-invasive inspection of contraband in vehicles, involves amplifying identical samples of x-ray beams using respective gain values, and forming x-ray image,” US Patent 2005047546-A1 (2005).

[6] Nittoh K. et al., “Extension of dynamic range in x-ray radiography using multi-color scintillation detector,” Nucl. Instrum. Methods Phys. Res. A 501(2–3), 615–622 (2003). 10.1016/S0168-9002(03)00424-8

[7] Chen P. Han Y. Pan J., “High-dynamic-range CT reconstruction based on varying tube-voltage imaging,” PLoS One 10(11), e0141789 (2015). POLNCL 1932-6203 10.1371/journal.pone.0141789 26544723 PMC4636367

[r8] Li Y. Han Y. Chen P., “X-ray energy self-adaption high dynamic range (HDR) imaging based on linear constraints with variable energy,” IEEE Photonics J. 10(2), 1–14 (2017). 10.1109/JPHOT.2017.2778719

[r9] Sisniega A. et al., “Dual-exposure technique for extending the dynamic range of x-ray flat panel detectors,” Phys. Med. Biol. 59(2), 421–439 (2014). PHMBA7 0031-9155 10.1088/0031-9155/59/2/421 24352046

[r10] Chen P. et al., “High-dynamic-range x-ray CT imaging method based on energy self-adaptation between scanning angles,” OSA Contin. 3(2), 253–266 (2020). 10.1364/OSAC.380634

[r11] Wei J. Han Y. Chen P., “Improved contrast of materials based on multi-voltage images decomposition in x-ray CT,” Meas. Sci. Technol. 27(2), 025402 (2016). MSTCEP 0957-0233 10.1088/0957-0233/27/2/025402

[r12] Qi Y. Yang Z. Kang L., “Multi-exposure x-ray image fusion quality evaluation based on CSF and gradient amplitude similarity,” J. X-Ray Sci. Technol. 29(4), 697–709 (2021). JXSTE5 0895-3996 10.3233/XST-210871 34057111

[13] Chen P. Han Y., “Varying-energy CT imaging method based on EM-TV,” Meas. Sci. Technol. 27(11), 114004 (2016). MSTCEP 0957-0233 10.1088/0957-0233/27/11/114004

[14] Haidekker M. A. et al., “Enhanced dynamic range x-ray imaging,” Comput. Biol. Med. 82, 40–48 (2017). CBMDAW 0010-4825 10.1016/j.compbiomed.2017.01.014 28160695

[15] Sukovic P. Clinthorne N. H., “A method for extending the dynamic range of flat panel imagers for use in cone beam computed tomography,” in IEEE Nucl. Sci. Symp. Conf. Record (Cat No. 01CH37310), IEEE (2001). 10.1109/NSSMIC.2001.1008657

[16] Münch B. et al., “Stripe and ring artifact removal with combined wavelet—Fourier filtering,” Opt. Express 17(10), 8567–8591 (2009). OPEXFF 1094-4087 10.1364/OE.17.008567 19434191

[17] Parker D. L., “Optimal short scan convolution reconstruction for fan beam CT,” Med. Phys. 9(2) 254–257 (1982). MPHYA6 0094-2405 10.1118/1.595078 7087912

[18] Edey D. et al., “Extending the dynamic range of biomedical micro-computed tomography for application to geomaterials,” J. X-Ray Sci. Technol. 27(5), 919–934 (2019). JXSTE5 0895-3996 10.3233/XST-190511 31356224

